# Peri-portal lymphedema in association with an acute adrenal insufficiency: case report

**DOI:** 10.1186/1752-1947-8-98

**Published:** 2014-03-24

**Authors:** Elamin Ibrahim Elamin Abdelgadir, Alaaeldin MK Bashier, Inas A Al Hameedi, Azza Abdulaziz, Sona Abuelkheir, Fatheya Alawadi

**Affiliations:** 1 Endocrine Unit, Dubai Hospital, Alkhaleej road, Dubai, P.O.BOX: 7272, United Arab Emirates; 2 Radiology Department, Dubai Hospital, Alkhaleej road, Dubai, P.O.BOX: 7272, United Arab Emirates

**Keywords:** Addison’s disease, Adrenal insufficiency, Crisis, Free water excretion, Lymphedema, Peri-portal lymphedema, Steroids

## Abstract

**Introduction:**

We report the case of a patient with peri-portal lymphedema in association with severe adrenal insufficiency. To the best of our knowledge, this association was not previously reported in the literature. Peri-portal lymphedema is usually seen in cases such as blunt abdominal trauma, hepatic congestion and post–liver transplantation.

**Case presentation:**

We present the case of a 28-year-old Indian man who presented to our hospital with adrenal crisis and was treated accordingly. Computed tomography of his abdomen showed evidence of peri-portal lymphedema (edema) with some free fluid collection. We excluded other causes of this pathology and followed the patient’s condition after steroid replacement therapy. We found no other contributing factors to the patient’s peri-portal lymphedema apart from the adrenal crisis, which was more consolidated when we followed the patient after steroid replacement therapy, during which follow-up computed tomography showed complete resolution of the pathology.

**Conclusions:**

We conclude following an extensive MEDLINE® search that this is the first case to be reported for the association between peri-portal lymphedema and adrenal insufficiency, after having excluded all other causes of peri-portal lymphedema. This signifies reporting of this case as the first one in the medical literature.

## Introduction

Acute adrenal insufficiency is a life-threatening medical condition. Treatment of acute adrenal insufficiency should not be delayed while making a definitive diagnosis.

It is always important to look for the underlying causes of adrenal insufficiency. Primary adrenal insufficiency could be due to autoimmune adrenalitis, a consequence of tuberculosis or adrenal gland hemorrhage. Less frequently seen causes include primary adrenal lymphoma [1] and X-linked adrenoleukodystrophy. Furthermore, many contributory genetic diseases might be included in the list of causes of adrenal insufficiency. In addition, adrenal insufficiency is rather common in the pediatric age group [2].

Adrenal insufficiency typically presents with vague symptoms with an insidious onset, such as fatigue, malaise, abdominal pain, weight loss, nausea and vomiting. This non-specific nature usually delays the diagnosis and raises the morbidity and/or mortality probability. Less common presentations have also been reported, such as serious neurological deterioration as a consequence of cerebral edema, which is thought to be due to severe hyponatremia [3]. Moreover, adrenal insufficiency has been shown to manifest, in a single case report, as an acute mesenteric ischemia [4], intestinal pseudo-obstruction and long-term intermittent hyponatremia [5].

Another rare presentation is the elevation in transaminases, which has been found to be related to adrenal insufficiency. There is no clear explanation for this association; however, different hypotheses have been set forth, including hepatic tissue hypoperfusion [6], autoimmune reaction toward the hepatic cells [6] and the possibility of hepatocyte apoptosis induced by local release of cytokines [6,7]. Olsson *et al*. reported that both alanine transaminase (ALT) and aspartate aminotransferase (AST) normalized within 1 week of replacement of steroids [8]. On the basis of that case, they suggested that Addison’s disease should be considered in the differential diagnosis of patients with unexplained elevated AST and ALT levels [8]. It is worth mentioning that relative adrenal insufficiency is common in patients with liver disease. It occurs in 33% of patients with acute liver failure and in 65% of those with chronic liver disease and sepsis [8,9].

## Case presentation

We report the case of a 28-year-old Indian man who has worked as a computer engineer in Dubai for the past 5 years. He presented to the Accident and Emergency Department of our hospital with complaints of lethargy, anorexia, nausea, vomiting and weight loss over a 1-month period. His symptoms had worsened over the preceding 4 days, during which time he became progressively unable to perform his daily activities until he was unable to get out of bed. He had normal bowel habits and no fever, night sweats or hair loss. He did not experience headaches or problems with vision, swallowing or hearing, nor did he complain of neurological weakness or numbness. He never complained of cough or hemoptysis. He had not had any recent trauma of any form.

Our patient was not known to have any previous medical problems and never had any infectious disorder, despite frequent visits to his home country. He was seen and assessed in many private hospitals, where he received intravenous fluids and vitamins and then discharged.

He was brought to the Emergency Room of our institution by ambulance. Our initial assessment was that he was a young man who was remarkably weak, emaciated, pale and severely dehydrated with sunken eyes. His physical examination results showed blood pressure 60/40mmHg, heart rate 100 beats/min, respiratory rate 18/min, O_2_ saturation 100% on room air, body temperature 36.5°C and blood glucose level 50mg/dL. During our systemic examination, we noted that he had hyperpigmented skin over his knuckles as well as in the oral mucosa. A chest examination revealed normal vesicular breathing with no added sounds. The cardiovascular, abdominal and locomotor system assessments did not show any abnormalities. Our neurological review showed that he was sleepy but arousable, his higher brain functions remained intact and he was moving all of his limbs, but with some limitation due to generalized weakness.

The patient received intravenous normal saline immediately. Dexamethasone was administered (after a sample of serum cortisol was taken). No inotropes were required. After 2 hours, he started to communicate perfectly, and his vital signs improved. His blood pressure had increased to 100/60mmHg, his heart rate was 86 beats/min, his respiratory rate was 16/min and his O_2_ saturation remained 100% on room air. The patient was admitted to the high-dependency medical ward with a provisional diagnosis of adrenocortical insufficiency and acute adrenal crisis. The results of our initial laboratory investigations are shown in Tables 1 and 2.

**Table 1:** **Initial biochemistry results**^**a**^

**Tests**	**Patient’s data**	**Units**	**Reference ranges**
Cortisol dynamic	1 (confirmed twice)	nmol/L	
ACTH	46.2	pg/mL	<46.0
Na^+^	134	mmol/L	136 to 145
K^+^	4.1	mmol/L	3.3 to 4.8
Cl^-^	112	mmol/L	98 to 108
Urea	52	mg/dL	12 to 40
Creatinine	2.4	mg/dL	0.7 to 1.2
Ca^2+^	9.3	mg/dL	8.9 to 10.2
Random glucose	119	mg/dL	
Magnesium	2.49	mg/dL	1.7 to 2.55
ALT	5	g/dL	0 to 41
AST	24	U/L	0 to 40
Alkaline phosphatase	53	U/L	40 to 129
Albumin	3.4	g/dL	3.4 to 4.8
Total bilirubin	0.3	mg/dL	0 to 1.0
Tissue transglutaminase	2.7	U/mL	<15
T4	10.6	pmol/L	11.5 to 22.7
T3	2.7	pmol/L	3.5 to 6.5
TSH	10.61	μIU/mL	0.55 to 4.78
ATG	82.5	IU/mL	<100
ATPO	670.7 (+ve)	IU/mL	<50
Anti-adrenal antibodies	Negative		<1:10
Blood culture	Negative		
Urine culture	Negative		
Stool culture	Negative		

^a^ACTH: Adrenocorticotropic hormone; ALT: Alanine transaminase; AST: Aspartate aminotransferase; ATG: Anti-thyroglobulin antibodies; TSH: Thyroid-stimulating hormone; ATPO: Anti-thyroid peroxidase antibodies (+ve ATPO indicates an autoimmunity related pathology that caused the hypothyroidism).

**Table 2:** **Initial hematology and serology**^**a**^

**Tests**	**Patient’s data**	**Units**	**Reference ranges**
WBCs	11.4	10^3^/μL	3.6 to 11.0
HGB	7	g/dL	13 to 18
MCV	83.7	fL	77 to 92
MCH	29.5	pg	26 to 34
MCHC	35.2	g/dl	32 to 36
RDW	14.5	%	11 to 14
PLT	177	10^3^/μL	150 to 400
PT	17.8	Seconds	11 to 14
aPTT	66	Seconds	28 to 41
INR	1.50	IU	
CRP	68	mg/L	<10
ESR	95	mm/hour	0 to 10
T-Spot TB test	Reactive		
Hepatitis B	Negative		
Hepatitis C	Negative		
HIV	Negative		
Anti-adrenal antibodies	Negative		<1:10
Stool for occult blood	Negative		

^a^aPTT: Activated partial thromboplastin time; CRP: C-reactive protein; ESR: Erythrocyte sedimentation rate; HGB: Hemoglobin; INR: International Normalized Ratio; MCH: Mean corpuscular hemoglobin; MCHC: Mean corpuscular hemoglobin concentration; MCV: Mean corpuscular volume; PLT: Platelets; PT: Prothrombin time; RDW: Red cell distribution width; TB: Tuberculosis; WBCs: White blood cells. HIV: Human immunodeficiency virus.

**Figure 1:**
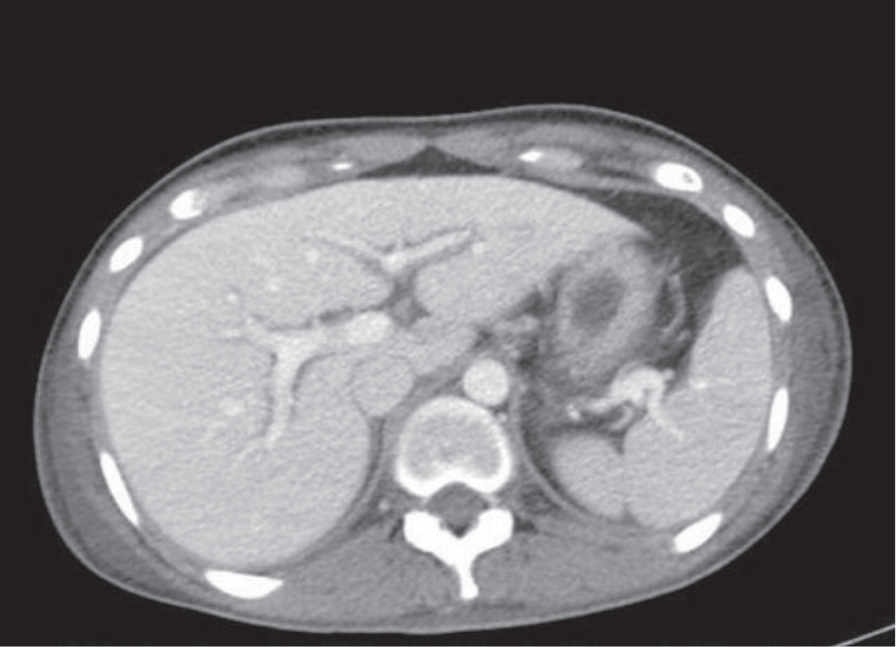
**Contrast-enhanced computed tomography scan showing axial section of the patient’s upper abdomen.** Note the peri-portal hypodensity at the level of the hilum, which is confined to the main portal veins and the first-order branches, consistent with peri-portal lymphedema.

His chest X-ray showed a faint patch of infiltrate in the right lower zone, and an air bronchogram was suggestive of interstitial pneumonitis (probably chronic), supported by the absence of clinical indicators of bacterial pneumonia. No pleural effusion was observed. An echocardiogram was taken by the emergency room physician, which showed no structural abnormalities, an ejection fraction of 55%, no wall motion abnormalities and no evidence of pulmonary embolism. Ultrasonography of the abdomen showed very minimal fluid collection at the hepatorenal pouch and no evidence of any free ascetic fluid collection at the pelvis. A computed tomography (CT) scan with contrast enhancement for the abdomen and pelvis showed an unremarkable appearance of the adrenal glands. We noticed hypodensity around the portal vein, suggestive of peri-portal lymphedema (edema). We also observed mild abdominal ascites and bilateral pleural effusion. A minimal amount of free fluid around the gallbladder and in the upper abdomen and pelvic areas (Figure 1) was seen.

The patient was continued on steroids and intravenous fluids. His condition improved substantially over a 10-day period. Meanwhile, we investigated the possible causes of his peri-portal edema and free fluid collection. No apparent reason was found, so we speculated that it could be associated with his adrenal insufficiency. Therefore, we decided that he could be discharged, with follow-up for this radiological feature after 8 to 12 weeks by obtaining another CT scan. At discharge, all of our patient’s blood tests had come down to normal, including the deranged coagulation profile, without any intervention. He showed significant clinical improvement, so he was discharged with prescriptions for hydrocortisone at a dosage of 20mg in the morning and 10mg in the evening and levothyroxine 25mg/day (to be started 2 weeks after the initiation of steroid replacement therapy), and we made an appointment for him to return to the endocrine clinic.

**Figure 2:**
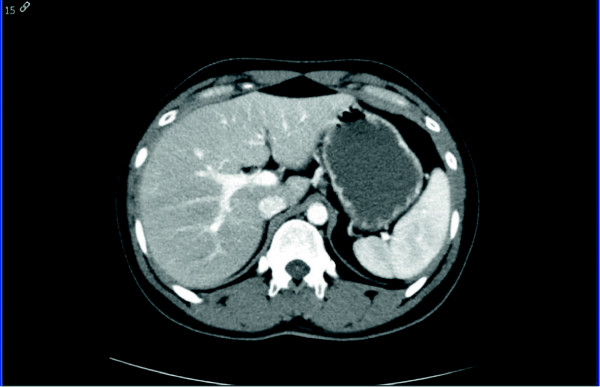
**Post-treatment follow-up computed tomography.** Scan shows complete resolution of the patient’s peri-portal lymphedema (lower panel).

At the patient’s follow-up visit at the clinic, he showed dramatic improvement in his general condition. CT scans of his abdomen showed complete resolution of the peri-portal lymphedema and the free fluid in the peritoneal and pleural spaces (Figure 2). A repeat chest X-ray did not show any significant changes from the previous study, consistent with non-specific interstitial changes. His follow-up laboratory blood test results are shown in Table 3.

**Table 3:** Follow-up results after 12 weeks

**Tests**	**Patient’s data**	**Units**	**Reference ranges**
Na^+^	134	mmol/L	136 to 145
K^+^	4.1	mmol/L	3.3 to 4.8
Cl^-^	112	mmol/L	98 to 108
Urea	52	mg/dL	12 to 40
Creatinine	2.4	mg/dL	0.7 to 1.2
Ca^2+^	9.3	mg/dL	8.9 to 10.2
WBCs	6.5	10^3^/μL	3.6 to 11.0
PT	12	Seconds	11 to 14
aPTT	32	Seconds	28 to 41
INR	1.10	IU	
ALT	11	g/dL	0 to 41
AST	22	U/L	0 to 40
Alkaline phosphatase	58	U/L	40 to 129
Albumin	3.9	g/dL	3.4 to 4.8
Total bilirubin	0.2	mg/dL	0 to 1.0
T4	16.2	pmol/L	11.5 to 22.7
T3	3.9	pmol/L	3.5 to 6.5
TSH	3.82	μIU/mL	0.55 to 4.78

Na: Sodium, K: potassium, Cl: chloride, Ca: Calcium, WBCs: White blood cells, aPTT: activated partial thromboplastin time, INR: International normalized ration, ALT: Alanine aminotransferase, AST: aspartae transaminase, T4: Thyroxine, T3: Triiodothyronine, TSH thyroid stimulating hormone.

## Discussion

Periportal lymphedema is a radiological finding of an intrahepatic periportal contrast enhancement. It is caused by impaired lymphatic drainage and peri-portal low attenuation corresponding to the numerous dilated lymphatic vessels and lymph congestion in the connective tissues around the portal vein and its branches [10,11].

Peri-portal lymphedema is related to the following:

1. Blunt abdominal trauma, especially if there is hepatic injury [12];

2. Lymphatic drainage jeopardy caused by either malignant lymphadenopathy (especially at the hepatoduodenal ligament) [10,11], surgical interruption [10], peri-portal infection or inflammation [10], liver cirrhosis, portal hypertension and Budd-Chiari syndrome [10];

3. Hepatic congestion in severe heart failure [13];

4. Post–liver transplantation [13]; and

5. Tumor infiltration and peri-portal fibrosis [10].

None of these conditions were relevant to the case of our patient. His lymphedema and free fluid collection had completely resolved after treatment with steroids was started, which suggested a causal relationship. We did an extensive MEDLINE® search to look for a co-relationship between Addison’s disease and peri-portal lymphedema. We used different search terms, such as “lymphedema”, “Addison” and “adrenal insufficiency”, but none of the results were relevant to our case. Therefore, to the best of our knowledge, our present case report is the first to describe an association between adrenal insufficiency and peri-portal edema.

A potential explanation of this phenomenon could be the association between adrenal insufficiency and impaired free water excretion. In their study published in 1962, Cutler *et al*. explored this association and concluded that the improvement in diuresis following glucocorticoid administration is due to direct effects on the renal tubules, as well as to improvement in renal hemodynamics [14]. In another study, published in 1964 by Kleeman *et al*., the authors described the association between antidiuretic hormones, adrenal insufficiency and free water excretion and concluded that adrenal insufficiency does not alter release, metabolism or action of anti-diuretic hormones [15]. However, they suggested that glucocorticoids have a direct effect on the permeability of certain membranes to water. This hypothesis could explain the free fluid accumulation, especially as it involves the most common areas for fluid accumulation in the body, namely, the pleural and peritoneal spaces.

## Conclusion

To the best of our knowledge, adrenocortical insufficiency has not previously been a known cause of peri-portal lymphedema (edema), and as such, we believe that our present case report is the first to describe such a causal relationship. The relation between peri-portal edema and the severity of disease needs to be evaluated further.

## Consent

Written informed consent was obtained from the patient for publication of this case report and any accompanying images. A copy of the written consent is available for review by the Editor-in-Chief of this journal.

## Abbreviations

ALT: Alanine transaminase; AST: Aspartate aminotransferase; CT: Computed tomography.

## Competing interests

The authors declare that they have no competing interests.

## Authors’ contributions

EA carried out the data collection of the case, conducted the follow-up in the outpatient department, wrote the manuscript draft and participated in sequence alignment. AB participated in sequence alignment, writing the manuscript, reviewing the literature and editing the manuscript. IA participated in writing the radiological comments and follow-up of the radiological images. SA participated in the sequence alignment and drafted the manuscript. AA participated in the sequence alignment, drafted the manuscript and participated in the literature review. FA supervised the case management. All authors read and approved the final manuscript.
